# Editorial: Development and neuroplasticity of the visual system, amblyopia and beyond

**DOI:** 10.3389/fnins.2026.1924302

**Published:** 2026-07-31

**Authors:** Kevin R. Duffy, Alex S. Baldwin, Anna Kosovicheva, Benjamin Thompson, Krista R. Kelly

**Affiliations:** 1 Department of Psychology and Neuroscience, Dalhousie University, Halifax, NS, Canada; 2 Department of Ophthalmology and Visual Sciences, McGill University, Montreal, QC, Canada; 3 Department of Psychological and Brain Sciences, University of Toronto, Mississauga, ON, Canada; 4 School of Optometry and Vision Science, University of Waterloo, Waterloo, ON, Canada

**Keywords:** amblyopia, development, neuroplasticity, vision, visual system

For over 60 years, the mammalian visual system has been a stalwart model for investigating experience-dependent brain development and plasticity. This includes seminal studies of visual deprivation (Wiesel and Hubel, 1963), critical periods (Olson and Freeman, 1980; Birch and Stager, 1996), and plasticity mechanisms (Heynen et al., 2003; Hensch, 2005), as well as contemporary investigations of neural reorganization and recovery (Echavarri-Leet et al., 2025; Birch et al., 2025; Bohnsack et al., 2026). Studies of the visual system have provided fundamental insights into how neural circuits are cultivated and impoverished by sensory experience. Research on amblyopia, a visual disorder that arises from abnormal development of neural connections, is particularly suited to expose meaningful characteristics of developmental neuroplasticity because its emergence and recovery are enabled by the pliability of neurons early in postnatal life.

The contributions in this Research Topic, *Development and neuroplasticity of the visual system, amblyopia and beyond*, present findings that span cellular, behavioral, and clinical disciplines. These studies reveal the breadth of contemporary visual neuroscience and highlight nascent topics that are reshaping the understanding of amblyopia and neural plasticity.

A central theme of this Research Topic is the recognition that amblyopia extends beyond a reduction in visual acuity. Several contributions examine the complex nature of amblyopia and the need to consider a broader constellation of perceptual and functional deficits. Molaei and Farivar make the case that perceptual distortions represent an important but underappreciated component of amblyopia, and advocate for a visual-field-wide and multi-feature-based approach to their characterization. They propose that distortions of form, position, orientation, and spatial organization may provide useful clues about the underlying neural dysfunction and possible treatment of amblyopia. Complementing this work, Goulet and Farivar synthesize evidence from human and animal studies to propose a dual-level neurophysiological framework for understanding amblyopic distortions. In this model, abnormalities in local feature encoding interact with altered spatial integration to produce the perceptual distortions experienced with amblyopia. These articles promote a more nuanced understanding of visual perception in amblyopia that acknowledge abnormalities beyond interocular suppression by detailing dysfunction across multiple levels of visual processing.

Another focus of this Research Topic is binocular vision and suppression. These phenomena are increasingly recognized as being central to both the pathophysiology and treatment of amblyopia. Hess provides a pertinent review of amblyopic suppression, examining why suppression occurs, how it is implemented within visual processing, and where it may arise within the visual pathway. This review reinforces the position that amblyopia is fundamentally a binocular disorder in which suppression is a central pathological mechanism, and that optimal treatment may require restoration of binocular cooperation rather than a primary focus on improving acuity in the impaired eye. In support of this view, Deng et al. demonstrate that short-term deprivation of the amblyopic eye can enhance binocular intermodulation responses measured using steady-state visual evoked potentials, a finding consistent with reduced interocular suppression. This demonstrates plasticity within binocular circuits that offers a possible electrophysiological biomarker relevant to treatment.

The translational importance of neuroplasticity is illustrated by two studies that examine recovery from amblyopia and visual deprivation. Using a model of deprivation amblyopia in cats, Mitchell et al. reveal that a prior unsuccessful treatment history can reduce the effectiveness of later therapeutic interventions, presumably by depleting plasticity resources in the visual system. Their findings spotlight the importance of visual experience history in determining recovery potential, and they underscore the value of early and enduring intervention. In animal models, temporary dominant-eye retinal inactivation has emerged as a novel and safe treatment for severe amblyopia (Duffy et al., 2018; Fong et al., 2021; Kwaw et al., 2026). De Paola et al. investigate the effects of monocular inactivation on neurofilament expression within the cat lateral geniculate nucleus. Their results reveal a sensitivity to retinal silencing outside the classical critical period, as well as a notable capacity for recovery following binocular vision restoration. These studies reveal that amblyopia treatment history can influence the capacity for visual plasticity, and also, that manipulation of retinal afferent activity at older ages can induce substantial structural plasticity in the visual system not previously observed.

This Research Topic highlights the importance of understanding biological mechanisms that regulate visual cortical plasticity. Murphy et al. review evidence supporting sex as a biological variable in amblyopia research and argue that similar behavioral outcomes may arise through divergent mechanisms in females and males. This contribution reflects a movement in neuroscience toward identifying factors that contribute to individual variability in developmental trajectories and treatment responses (Jia et al., 2022). At the cellular level, Panday et al. provide a high-dimensional analysis of parvalbumin-positive interneuron morphology in visual cortex. Because parvalbumin interneurons are important regulators of critical period plasticity through cortical inhibition (Hensch and Quinlan, 2018; Huang et al., 2024), this research offers a valuable anatomical framework for studies investigating how inhibitory circuits shape experience-dependent neural reorganization of the visual system.

Complementing traditional measures of vision, several contributions examine how amblyopia affects everyday function. Dranitsaris and Reynaud review the impact of amblyopia and binocular vision deficits on reading performance, presenting evidence for altered eye movements and reduced reading speed, while pointing out educational and social consequences. This work reveals the significant detrimental impact of amblyopia on life quality that extends past clinical measurements of vision. Granados-Delgado et al. investigate the relationship between binocular visual function and eye-hand coordination in healthy subjects. They provide evidence that individual differences in binocular processing, such as phoria, can significantly impact reaching performance. Nasir et al. present findings that adult humans with unilateral deprivation amblyopia exhibit persistent impairments in fixation stability, indicating that early visual deprivation can produce durable alterations in oculomotor control.

The articles in this Research Topic reveal a diverse and evolving field. Amblyopia is no longer viewed solely as a disorder of visual acuity, nor is the window of neuroplasticity thought to close permanently after early postnatal development. These contributions demonstrate that alterations of early visual experience can effect profound change across molecular pathways, eye movements, visual behavior, and perception. Notwithstanding the substantial developmental disruptions occasioned by amblyogenic factors, recovery remains possible when the mechanisms of neuroplasticity can be resourced. Improvements in vision, for instance, have recently been documented outside the critical period following regimes of binocular (Ding et al., 2026) or amblyopic-eye (Lin et al., 2026) perceptual training. Within the critical period, a novel asynchronous dichoptic therapy that imposes a temporal offset in stimulating the amblyopic eye before the fellow eye has shown superior efficacy relative to patching in improving visual acuity and stereoacuity (Wen et al., 2026; Gaier et al., 2026).

We hope this Research Topic of papers inspires further investigation into the factors that govern visual system function, development, and plasticity. Importantly, many of the themes explored in this Research Topic extend beyond amblyopia. The pursuit of a deeper understanding of critical periods, inhibitory regulation, circuit reorganization, and recovery after altered experience is a fundamental challenge that transcends amblyopia and visual system plasticity. We are optimistic that insights gained from this Research Topic will canalize future research by encouraging varied perspectives across fundamental and clinical science. In this way, we can advance the understanding and treatment of amblyopia while discovering broader principles of brain development, structure and function.
